# Change in physical workload and the risk of work disability due to musculoskeletal disorders: a register-based study of 1 703 935 workers in Sweden

**DOI:** 10.1136/oemed-2026-111034

**Published:** 2026-07-28

**Authors:** Daniel Falkstedt, Tomas Hemmingsson, Laura Punnett, Katarina Kjellberg, Alicia Kurowski, Kuan-Yu Pan, Melody Almroth

**Affiliations:** 1 Institute of Environmental Medicine, Karolinska Institutet, Stockholm, Sweden; 2 Department of Public Health Sciences, Stockholm University, Stockholm, Sweden; 3 Department of Biomedical Engineering, University of Massachusetts, Lowell, Massachusetts, USA; 4 Centre for Occupational and Environmental Medicine, Region Stockholm, Stockholm, Sweden

**Keywords:** Sickness absence, Physical Exertion, Musculoskeletal System

## Abstract

**Objective:**

This register-based study examined whether changes over 14 years in occupational physical workload (PWL) affected the risk of registered work disability due to a musculoskeletal disorder (MSD).

**Methods:**

This study included 1 703 935 workers in Sweden in 2005. PWL exposure was measured using job-exposure matrices linked to registered occupations in 1990 and 2005; change in PWL was defined between categories (low, medium, high) in either direction. The outcome was cumulative incidence of ‘work disability’ (either long-term sickness of at least 2 years or disability pension) due to MSD from 2006 to 2019. Crude and multivariate Cox proportional hazards regression models estimated sex-specific HRs with 95% CIs. Models were adjusted for sociodemographic factors and health and employment history.

**Results:**

A total of 33 369 individuals (1.96%) became work disability cases during follow-up. Compared with workers with consistently high PWL, any reduction in PWL was associated with a decreased risk of work disability due to MSD (women: HR 0.57, 95% CI 0.52 to 0.63, men: HR 0.59, 95% CI 0.52 to 0.66 for change from high to low exposure). Compared with workers with consistently low PWL, any increase was associated with a higher risk of work disability due to MSD (women: HR 1.68, 95% CI 1.53 to 1.86, men: HR 2.19, 95% CI 1.89 to 2.52 for change from low to high).

**Conclusion:**

In both sexes, decreases in PWL exposure were associated with a lower risk of MSD disability and increases in exposure with a higher risk.

**WHAT IS ALREADY KNOWN ON THIS TOPIC**

- While there is an established link between heavy physical workload and musculoskeletal work disability, few studies have investigated whether a change in exposure level could lead to a different work disability risk.

**WHAT THIS STUDY ADDS**

- Using population data and occupational-level measures measured 16 years apart, we found that in both men and women, changing from higher to lower exposure to physical workload was associated with a lower risk of work disability due to a musculoskeletal disorder, and changing from lower to higher exposure was associated with higher risks.

**HOW THIS STUDY MIGHT AFFECT RESEARCH, PRACTICE OR POLICY**

- Reducing life-course exposure to heavy physical workload could improve people’s labour market prospects associated with musculoskeletal health and work ability.

## Introduction

 Musculoskeletal disorders (MSDs) are a common reason for absences from work and premature exit from the labour market.^1^ There is consistent evidence for a relationship between heavy occupational physical workload (PWL) and an increased risk of MSD work disability,^2–8^ such as registered sickness absence or disability pension.

Few studies have examined whether changes in PWL predict later changes in MSD risk. Halonen *et al*^9^ found that those with high PWL in young adulthood whose exposures were lower in older adulthood had a lower risk of diagnosed MSDs compared with those with consistently high PWL. Similarly, Badarin *et al*^10^ found that men and women who changed from higher to lower occupational PWL during a 5-year period reduced their risk of MSD disability pension. However, Cillekens *et al* found that increase in physical strain at work was followed by a higher risk of MSD symptoms within a 1-year period among women only, while decreased strain showed no clear benefit for either gender.^11^

Changes in PWL have also been related to broader health and work outcomes. Studies from Finland^12^ and the Netherlands^13^ found that increases in PWL were associated with poorer health functioning and reduced self-reported work ability, respectively, whereas decreases in PWL had more favourable results, although less consistently. Similarly, reductions in PWL have been associated with lower risks of sickness absence,^14^ all-cause disability pension^10^ and labour market exit.^15^

Some variation in the cited findings may be due to differences in outcome measure, design and operationalisation of change. The use of self-report^11–13^ might introduce systematic bias if PWL is experienced differently based on pre-existing MSD difficulties. Some studies report only pooled results for men and women,^9 12 13^ even though men and women often work in different sectors of the labour market and may be differentially exposed to PWL even within the same occupations.

The present study extends the limited evidence in several ways. First, we use more objective information on PWL derived from job-exposure matrices (JEMs). Second, our MSD outcome information is based on diagnoses from medical professionals. Third, our exposure measure has sufficient range to permit three levels, so that we can examine both increases and decreases from baseline values. Fourth, our large dataset allows us to analyse men and women separately. Fifth, the study is register-based with very limited attrition and covers all occupations on the labour market.

The aim of this study was to examine whether an increase or decrease in occupational PWL prospectively affects the risk of MSD work disability. Our hypotheses were that a decreased PWL would lower the risk of MSDs and increased PWL would increase the risk, independent of the initial level of PWL.

## Methods

### Study population

This study used data from the Swedish Work, Illness and labour-market Participation cohort, created through linkages of national registers using unique personal identity numbers for persons registered as living in Sweden,^16^ including all individuals aged 16–64 years in 2005 (≈5.8 million).

**Figure 1:**
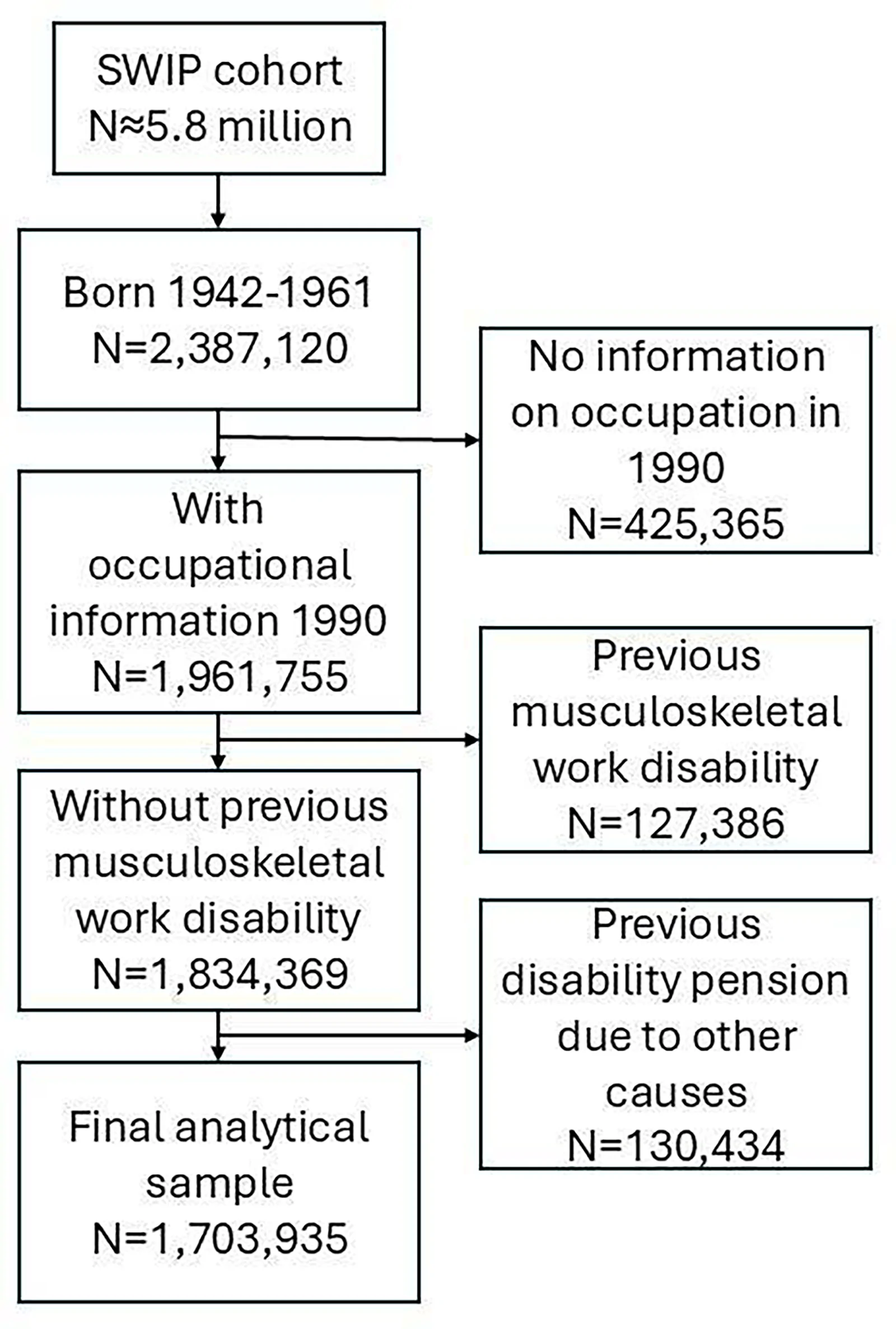
Flow chart of source population and analytical sample. SWIP, Swedish Work, Illness and labour-market Participation.

The initial sample included 2 387 120 individuals who were born between 1942 and 1961, as those aged 29–48 years in 1990 were likely established in their jobs in 1990 and still active in the labour market in 2005, at a maximum age of 63 years. We excluded 425 365 individuals with no information on initial PWL in 1990, 127 386 individuals with disability pension or long-term sickness absence due to MSDs (our operationalisation of work disability) prior to 2006 and another 130 434 with full-time disability pension due to other causes. The final analytical sample was 1 703 935 individuals followed from 1 January 2006 to 31 December 2019 (figure 1).

### Exposure

PWL was measured using JEMs and linked to the participants’ registered occupations in 1990 and 2005 (information was not registered or was incomplete for the years in-between). The JEMs were constructed based on data from the Swedish Work Environment Surveys conducted every second year between 1989 and 2013.

Due to a change in occupational coding systems, two JEMs were used. PWL in 1990 was estimated from worker survey responses from 1989 to 1997, providing mean PWL index values by sex for 320 occupations coded according to the 1983 version of the Nordic Occupational Classification system. PWL in 2005 was estimated from a survey covering 1997–2013, providing sex-specific mean values for 355 occupations coded according to the Swedish Standard Classification of Occupation.

Both JEMs are derived from the same eight questions regarding physical loads including heavy lifting, uncomfortable working postures, repetitive work and physically demanding work.^10^ Sex-specific mean index values were linked to individuals based on their occupational titles in 1990 (from the Swedish census) and 2005 (from the national occupations register).

Tertiles of PWL (low, medium, high) were defined separately for men and women in 1990 and 2005. Change in exposure was categorised as staying at the same exposure level, increasing by one or two levels, decreasing by one or two levels or missing occupation in 2005.

### Outcome

MSD work disability was operationalised as incident cases of full-time or part-time long-term sickness absence of at least 2 years or disability pension due to any diagnosis in the M chapter of the International Classification of Diseases, Tenth Revision (ICD-10), occurring from 2006 to 2019. This information was obtained from the Micro Data for Analysis of the Social Insurance System register. In Sweden, diagnoses of sickness absence and disability pension are based on ICD codes provided in physicians’ certificates, which constitute the medical basis for decisions regarding eligibility for benefits. For disability pension, a comprehensive certificate assessing diagnosis and work capacity is required. Thus, diagnostic information is derived from clinical assessments rather than self-report.

Individuals aged 30–64 years whose injuries, illnesses or disabilities prevent them from working are eligible to collect disability pension in Sweden. We included very long-term sickness absence in our definition of work disability because it arguably represents a severe MSD-related labour market outcome, in line with disability pension. As other social insurance systems in other contexts may not differentiate between these types of outcomes, this also makes our study more comparable with others.

### Covariates

Several sociodemographic, labour-market-related and health-related covariates were chosen a priori based on their potential to act as confounders (being associated with both accumulated PWL and later MSD work disability). Because the exposure period covers a 16-year window, we prioritised variables likely to be established prior to labour market entry, as well as indicators of labour market marginalisation during the exposure period, while avoiding adjustment for factors that might lie on the causal pathway.

Information obtained from the Longitudinal Integrated Database for Health Insurance and Labour Market Studies (LISA)^17^ register in 2005 included age, highest attained education, marital status and birth country. Age was categorised into three levels for descriptive analyses (44–49, 50–59 and 60–64 years); education was categorised into five levels indicating total years of schooling (≤9, 10–11, 12, 13–14 and >15) ranging from compulsory school only to university education; marital status was categorised as never married, married, divorced and widowed and birth country was dichotomised as Sweden or other. Information on parents’ socioeconomic position (SEP) (a proxy for early-life socioeconomic circumstances) was obtained from population censuses and categorised into unskilled manual, skilled manual, assistant non-manual, intermediate non-manual, higher non-manual, farmer and no registered occupation.

To account for health-related selection and labour-market attachment during the exposure period, we included previous part-time disability pension due to other causes, total unemployment days 10 years before 2006 (1996–2005) and total sick leave days before 2006 (1996–2005), drawn from the LISA register and categorised as 0, 1–365, 366–730 and >730 days.

### Statistical analysis

The distribution of covariates was explored based on PWL levels in 1990 and 2005. Sex-stratified Cox proportional hazard models were run for each covariate to estimate HRs and 95% CIs for associations with MSD work disability from 2006 to 2019. Individuals were censored at death, emigration, age 65 years or end of follow-up (31 December 2019), whichever came first. Information on death and migration was obtained from the total population register.^18^

Multivariable models examined change in PWL from 1990 to 2005 and risk of MSD work disability. Age was used as the underlying timescale. Models were stratified based on PWL in 1990, with those stable in low, medium or high exposure between 1990 and 2005, respectively, used as reference categories, and run separately for men and women. Model 1 was adjusted for age, education, marital status, country of birth and early-life SEP. Model 2 was adjusted for all variables included in model 1 and previous part-time disability pension due to other causes, unemployment days from 1996 to 2005 and sickness absence days from 1996 to 2005.

To more closely assess the importance of age, models were stratified by age category in 1990.

All analyses were conducted using STATA V.17 (StataCorp, College Station, Texas, USA).

## Results

Compared with those who stayed at the same exposure level, men and women whose PWL increased from 1990 to 2005 tended to be lower educated, more likely to be born outside of Sweden, have a lower childhood SEP and more previous days on unemployment and sickness absence (table 1 and online supplemental table S1). People whose PWL decreased tended to be younger, more highly educated, less likely to be born outside of Sweden and had a higher childhood SEP. Women whose exposure level increased were more likely to be unmarried.

**Table 1:** Characterisation of the cohort according to select covariates in relation to occupational PWL measured in 1990 and 2005

PWL 1990	Low				Medium				High			
**PWL 2005**	**Low**	**Medium**	**High**	**Missing**	**Low**	**Medium**	**High**	**Missing**	**Low**	**Medium**	**High**	**Missing**
Women, n (%)	222 531 (77.0%)	36 254 (13.0%)	15 304 (5.3%)	14 904 (5.2%)	93 610 (35%)	125 590 (46%)	36 293 (13%)	15 109 (5.6%)	24 806 (10%)	35 249 (15%)	161 302 (66%)	21 549 (8.9%)
Age (years)												
44–49	23.7	25.8	27.9	21.4	31.6	31.9	33.7	24.5	46.6	38.4	30.2	28.1
50–59	54.9	54.8	53.9	46.5	52.2	52.0	50.0	44.8	43.6	46.8	50.8	47.2
60–64	21.4	19.4	18.2	32.1	16.2	16.1	16.3	30.7	9.8	14.8	19.0	24.7
Previous DP, other causes	4.0	4.2	4.0	4.7	3.3	4.3	4.2	4.8	4.0	3.8	4.2	5.6
Unemployed days, 1996–2005												
0	83.4	73.3	46.5	54.2	79.1	83.6	57.5	57.6	60.0	63.5	74.7	58.8
1–365	10.4	15.6	28.3	16.0	13.6	11.4	26.1	16.5	24.1	22.7	15.6	15.3
366–730	3.8	6.3	13.7	12.1	4.6	3.2	9.8	11.2	9.4	8.3	5.6	10.7
>730	2.4	4.8	11.5	17.7	2.7	1.8	6.6	14.7	6.5	5.5	4.1	15.2
Sick leave days, 1996–2005												
0	49.6	42.9	34.1	48.8	47.8	40.4	32.4	45.5	40.9	37.7	32.9	43.1
1–365	39.5	45.1	52.5	38.1	41.3	46.4	53.3	40.9	45.7	49.2	54.0	43.6
366–730	5.2	5.9	7.1	5.6	5.5	6.3	7.7	6.0	6.7	6.9	7.3	6.1
>730	5.7	6.1	6.3	7.5	5.4	6.9	6.5	7.6	6.7	6.2	5.8	7.2
Men, n (%)	233 647 (75%)	38 687 (12%)	16 781 (5.4%)	23 287 (7.3%)	84 333 (29%)	113 163 (39%)	59 739 (20%)	35 308 (12%)	22 837 (7.7%)	48 559 (16%)	178 560 (60%)	46 533 (16%)
Age (years)												
44–49	25.0	24.8	24.1	17.7	30.9	28.9	33.1	26.0	42.7	36.5	32.5	29.9
50–59	52.9	54.0	53.5	49.2	51.6	52.4	50.6	48.8	45.6	49.0	50.8	49.7
60–64	22.1	21.2	22.4	33.1	17.5	18.7	16.3	25.2	11.7	14.5	16.7	20.4
Previous DP, other causes	1.2	2.4	1.9	2.8	1.4	2.2	2.3	3.2	1.5	2.7	2.3	3.4
Unemployed days, 1996–2005												
0	87.6	75.6	63.7	63.8	81.6	80.4	69.0	57.6	69.7	66.5	67.6	59.1
1–365	7.7	13.2	18.4	14.1	11.2	11.2	16.0	16.4	17.6	16.9	16.1	15.4
366–730	2.9	5.8	9.1	9.0	4.3	4.6	7.4	10.7	6.9	8.2	7.7	9.5
>730	1.8	5.4	8.8	13.1	2.9	3.8	7.6	15.3	5.8	8.4	8.6	16.0
Sick leave days, 1996–2005												
0	72.6	58.8	51.2	60.8	67.1	54.7	47.5	52.0	58.5	47.3	45.2	44.5
1–365	23.3	34.0	41.0	30.8	27.9	38.3	44.7	37.2	34.2	43.9	47.0	44.1
366–730	2.2	3.9	4.6	3.8	2.8	3.9	4.6	5.1	4.1	5.1	4.7	5.9
>730	1.9	3.3	3.2	4.6	2.2	3.1	3.2	5.7	3.2	3.7	3.1	5.5

DP, disability pension; PWL, physical workload.

A total of 33 369 individuals (1.96%) developed new MSD work disability during the follow-up period. As the outcome was rare, the total person-time at risk in the cohort was close to the total number of people at risk multiplied by 14 years of observation.

For both men and women, dose-response relationships were evident: there was higher risk of MSD-related work disability with higher age, lower level of education and lower parental SEP (table 2). Additionally, divorced and widowed and foreign-born men and women, as well as those with previous part-time disability pension, were all significantly more at risk of work disability during follow-up.

**Table 2:** Associations between each covariate and work disability due to a musculoskeletal disorder

	Women	Men
Total number of cases	20 263 (2.5%)	13 106 (1.5%)
Covariates		
Age (years)	HR (95% CI)	HR (95% CI)
44–49	1	1
50–59	2.57 (2.46 to 2.67)	2.42 (2.30 to 2.55)
60–64	12.33 (11.59 to 13.12)	10.69 (9.92 to 11.52)
Education (years)		
>15	1	1
13–15	1.57 (1.47 to 1.67)	1.94 (1.74 to 2.17)
12	1.93 (1.81 to 2.06)	3.41 (3.08 to 3.76)
10–11	3.09 (2.94 to 3.25)	5.18 (4.73 to 5.66)
≤9	4.51 (4.27 to 4.76)	7.48 (6.83 to 8.18)
Marital status		
Married	1	1
Unmarried	0.87 (0.84 to 0.90)	1.00 (0.96 to 1.04)
Divorced	1.35 (1.30 to 1.39)	1.34 (1.28 to 1.40)
Widowed	1.64 (1.51 to 1.77)	1.91 (1.66 to 2.20)
Foreign born	1.59 (1.53 to 1.66)	1.85 (1.76 to 1.94)
Parents’ occupation		
Higher non-manual	1	1
Intermediate non-manual	1.33 (1.21 to 1.46)	1.39 (1.22 to 1.59)
Assistant non-manual	1.51 (1.37 to 1.66)	1.58 (1.38 to 1.82)
Farmer	1.91 (1.73 to 2.11)	2.60 (2.27 to 2.97)
Skilled manual	2.09 (1.91 to 2.28)	2.69 (2.37 to 3.05)
Unskilled manual	2.31 (2.11 to 2.52)	2.92 (2.58 to 3.30)
No information	3.07 (2.80 to 3.36)	4.23 (3.72 to 4.80)
Previous DP due to other causes	1.39 (1.30 to 1.48)	1.40 (1.24 to 1.58)
Unemployment days (1996–2005)		
0	1	1
1–365	1.35 (1.30 to 1.40)	1.77 (1.69 to 1.85)
366–730	1.79 (1.70 to 1.88)	2.28 (2.15 to 2.41)
>730	2.29 (2.17 to 2.41)	2.97 (2.82 to 3.13)
Sick leave days (1996–2005)		
0	1	1
1–365	4.16 (4.00 to 4.35)	4.87 (4.64 to 5.11)
366–730	12.70 (12.10 to 13.40)	16.52 (15.57 to 17.52)
>730	14.0 (13.3 to 14.8)	19.60 (18.47 to 20.81)

DP, disability pension.

Men and women with higher initial PWL in 1990 were more likely to develop work disability during follow-up (table 3). For both men and women, increasing PWL exposure from 1990 to 2005 was associated with higher risk of work disability. For those with *low PWL in 1990*, the risk for work disability was higher among those who changed to moderate (women: HR 1.67, 95% CI 1.54 to 1.82; men: HR 3.07, 95% CI 2.73 to 3.46) or high (women: HR 3.01, 95% CI 2.75 to 3.31; men: HR 5.60, 95% CI 4.91 to 6.38) PWL in 2005, compared with those who remained in the low PWL category. For those in the *medium category in 1990*, increased PWL was associated with higher risk (women: HR 1.74, 95% CI 1.63 to 1.85; men: HR 1.54, 95% CI 1.43 to 1.65) and decreased PWL with lower risk (women: HR 0.61, 95% CI 0.57 to 0.65; men: HR 0.48, 95% CI 0.43 to 0.53), compared with those stable at moderate exposure. Among those with *high PWL in 1990*, there was a protective effect as they moved to moderate (women: HR 0.68, 95% CI 0.64 to 0.72; men: HR 0.76, 95% CI 0.71 to 0.81) or low (women: HR 0.43, 95% CI 0.39 to 0.46; men: HR 0.47, 95% CI 0.42 to 0.53) exposure to PWL in 2005 compared with those stable in the high exposed category. Adjustment for covariates substantially attenuated the associations, especially among those who changed from low to higher exposure, and especially when including previous days of sickness absence, disability pension and unemployment.

**Table 3:** Association between levels of PWL in 1990 and 2005 and risk of work disability due to MSD among women and men

PWL 1990	MSD %	PWL 2005	MSD %	Crude	Adjusted 1	Adjusted 2
				HR (95% CI)	HR (95% CI)	HR (95% CI)
**Women**						
Low	1.4	=	1.1	1	1	1
		+1	2.0	1.67 (1.54 to 1.82)	1.55 (1.42 to 1.68)	1.35 (1.24 to 1.47)
		+2	3.6	3.01 (2.75 to 3.31)	2.32 (2.10 to 2.55)	1.68 (1.53 to 1.86)
		Missing	2.1	2.14 (1.91 to 2.41)	1.93 (1.72 to 2.18)	1.36 (1.20 to 1.54)
Medium	2.4	=	2.4	1	1	1
		−1	1.5	0.61 (0.57 to 0.65)	0.66 (0.62 to 0.70)	0.72 (0.67 to 0.77)
		+ 1	4.2	1.74 (1.63 to 1.85)	1.37 (1.29 to 1.46)	1.20 (1.13 to 1.29)
		Missing	3.0	1.52 (1.38 to 1.68)	1.35 (1.22 to 1.50)	1.18 (1.06 to 1.30)
High	4.0	=	4.5	1	1	1
		−1	3.3	0.68 (0.64 to 0.72)	0.78 (0.73 to 0.83)	0.78 (0.73 to 0.83)
		−2	2.3	0.43 (0.39 to 0.46)	0.57 (0.52 to 0.63)	0.57 (0.52 to 0.63)
		Missing	3.7	0.89 (0.83 to 0.96)	0.92 (0.85 to 0.99)	0.88 (0.81 to 0.95)
**Men**						
Low	0.6	=	0.3	1	1	1
		+1	1.1	3.07 (2.73 to 3.46)	2.22 (1.96 to 2.51)	1.66 (1.46 to 1.88)
		+2	1.9	5.60 (4.91 to 6.38)	3.37 (2.93 to 3.88)	2.19 (1.89 to 2.52)
		Missing	1.0	3.34 (2.88 to 3.88)	2.72 (2.34 to 3.16)	1.66 (1.42 to 1.95)
Medium	1.4	=	1.4	1	1	1
		−1	0.7	0.48 (0.43 to 0.53)	0.57 (0.52 to 0.63)	0.68 (0.62 to 0.75)
		+1	2.2	1.54 (1.43 to 1.65)	1.32 (1.22 to 1.42)	1.18 (1.10 to 1.28)
		Missing	2.1	1.66 (1.52 to 1.81)	1.58 (1.44 to 1.72)	1.18 (1.08 to 1.30)
High	2.5	=	2.6	1	1	1
		−1	2.0	0.76 (0.71 to 0.81)	0.78 (0.72 to 0.83)	0.77 (0.71 to 0.82)
		−2	1.3	0.47 (0.42 to 0.53)	0.54 (0.48 to 0.61)	0.59 (0.52 to 0.66)
		Missing	2.8	1.15 (1.08 to 1.22)	1.15 (1.08 to 1.22)	0.96 (0.91 to 1.03)

Those stable at each exposure level (=) from 1990 to 2005 are the reference category. HRs and 95% CIs.

=unchanged; −1 decreased by one level; −2 decreased by two levels; +1 increased by one level; +2 increased by two levels.

Adjusted 1: age, education, marital status, country of birth, early-life SEP.

Adjusted 2: adjusted 1+previous DP due to other causes, unemployment days 1996–2005, sickness absence days 1996–2005.

DP, disability pension; MSD, musculoskeletal disorder; PWL, physical workload; SEP, socioeconomic position.

For both men and women, moving from low PWL in 1990 to missing occupational information in 2005 was associated with an increased risk of work disability, while moving from high PWL to missing was associated with a decreased risk. Moving from medium PWL in 1990 to missing in 2005 was not associated with any change in work disability after adjustments.

In the models stratified by age (categorised as 29–34, 35–44 and 45–49 years in 1990), associations between increases and decreases in PWL and MSD work disability were found across age groups (online supplemental table S2). However, associations tended to be stronger among the oldest group, especially for women.

## Discussion

This population-based study found that workers who changed from occupations with high or moderate PWL to lower PWL had a lower risk of MSD work disability (disability pension or >2 years of sickness absence), compared with those whose exposure remained higher. Increasing PWL was associated with a higher risk than remaining at lower levels. Associations were attenuated after controlling for education, prior unemployment, sickness absence and sociodemographic factors, especially among those moving from low to higher PWL.

Previous research has highlighted uncertainty as to whether the effect of changes in PWL depends on prior exposure level.^19^ We stratified analyses by baseline exposure, showing internally consistent results: an increase of one exposure category had a similar effect regardless of initial level (except for men starting at low exposure who showed a larger increased risk), while decreases had comparable effects across groups. Age-stratified analyses showed that associations were consistent across age groups, and possibly suggest stronger associations among the oldest workers.

### Comparison with other studies

Only three previous studies have investigated change of exposure to PWL and risk of MSDs. Cillekens *et al*^11^ found that an increased level of self-reported heavy workload within a 1-year period was associated with increased MSD symptoms among women but found no association for reduced PWL. The authors speculated that, as MSDs often become chronic and long-lasting, the short follow-up period made it less likely that a change to lower workload could result in reducing symptoms. The present results were obtained with 14 years of follow-up, indicating that it could require a longer period for MSD risk to abate after a reduction in PWL. Halonen *et al*^9^ found that those reporting to be exposed to high PWL in young adulthood, but not 20 years later showed a higher risk of diagnosed MSDs compared with those with stable low PWL. Those with high exposure reported in both surveys showed an even higher risk. Both Cillekens *et al* and Halonen *et al* analysed two levels of exposure only (high or low) while the present study used three exposure groups.

Using the same cohort as the present study, Badarin *et al*^10^ showed that older Swedish workers who changed from high to moderate or low PWL over 5 years had a lower risk of MSD disability pension than those without such an exposure decrease. Changes in other directions were not investigated. The present study extends those results with a longer period between measures of exposures and a wider age range of workers initially exposed to high PWL. Due to the longer observation period, more people changed from high to lower exposure in the present study (25%) than in the study by Badarin *et al* (4%).

Strengths of this study include the use of national register data minimising the risk of selection (related to attrition) and information bias (related to self-report). Including both MSD-sickness absence of at least 2 years and disability pension in the outcome definition made the results more generalisable to other contexts. The size of the study population, consistency of information quality and availability of a common exposure metric for all occupations enabled analyses of multiple magnitudes and direction changes in exposure levels over time, conditional on the initial exposure level, in women and men separately.

Compared with self-reported information, the use of JEMs reduces the likelihood of differential misclassification and spurious risk estimates,^20^ but it also has limitations. Differences in exposure between individuals within the same occupation are eliminated and are likely to result in non-differential misclassification and downward bias of risk estimates. This means that we only measured changes in PWL due to changes of occupation or changes in the occupational classification (potentially due to improved routines and equipment between 1990 and 2005), but not due to individual-level changes through changing work tasks or place of employment within the same occupational title. The JEMs used for 1990 and for 2005 were based on different occupational coding systems, which may have led to exposure misclassification if some changes in PWL were not actual changes, but rather a different classification of the same occupation. This would be more likely for a one-unit change than a two-unit change and would likely lead to an underestimation of the risk.

We lacked information on lifestyle factors important for MSDs such as leisure-time physical inactivity or obesity. However, we adjusted for level of education, which is associated with health habits.^21^ Another important source of potential confounding is mental health problems, which often co-occur with MSDs. For the period prior to baseline, only inpatient records were available, meaning only severe mental health problems resulting in hospitalisation would have been included. Instead, we adjusted for previous sickness absence and part-time disability pension, which partially cover health and labour-market vulnerabilities. We did not adjust for psychosocial occupational characteristics, as these are also measured with JEMs and are highly correlated with PWL. As with all observational studies, other residual confounding cannot be ruled out.

We lacked information on timing of changes in PWL during the exposure period and therefore cannot confirm the temporal sequence of exposure change and MSD health during the observation period. We stratified analyses by age to better understand if associations were present across age groups, but since we lacked more specific information about age of transition, it was difficult to further interpret these patterns. Those who changed from low to higher exposure to PWL had weaker labour-market attachment, with more days of unemployment and sick leave during the exposure period (table 1). Whether these events were a consequence of increased exposure or preceded it in some causal way is unknown. Thus, there may have been some overadjustment resulting from inclusion of prior disability and absenteeism in the models.

Selection effects, including the healthy worker effect, may have led to an underestimation of estimates. Those who did not have a registered occupation in 1990 and those who were on disability pension prior to 2005 were excluded, even though they could have already left the labour market because of their working conditions. We retained those who did not have a registered occupation in 2005 to understand the patterns of those who had left the labour market during the exposure period. This group tended to have a higher risk of work disability regardless of their starting exposure, except those who started out highly exposed. While this indicates that this group was marginalised, it is difficult to further disentangle, as the reasons for not having a registered occupation are diverse. Similarly, we cannot disentangle the reasons for a change in occupation. For example, reducing exposure through job change could indicate a more positive trajectory that could represent other innate advantages and benefits or conversely, a reduction in PWL could be due to poor health and work ability.

### Summary and interpretation of results

This study confirms the dose-response relationship between PWL and MSD work disability. Most importantly, we found that changes from high to lower exposure to PWL were associated with lower risk, and changing from low to higher PWL was associated with a higher risk, compared with those who remained at higher or lower exposure levels. Among those who started with moderate exposure, changing to higher exposure increased the risk and changing to lower exposure decreased the risk. These changes appeared particularly important among older workers, who may be especially vulnerable to the effects of high PWL. This knowledge is useful for employers and employees, as reducing exposure by any amount may lower the risk of labour-market exit due to MSDs.

Although the findings may be partly specific to the Swedish welfare context, they likely capture more general relationships between long-term PWL decreases or increases and trajectories of MSD problems, resulting in labour-market implications across different settings. If the observed associations represent underlying causal effects of workload reductions (or increases) on health and work ability, then the results are relevant to other contexts. However, replication studies are encouraged.

Although we could not disentangle the mechanisms behind changes in exposure, this study strengthens the evidence for a causal association between PWL and MSD work disability. Several key epidemiological criteria for causal inference^22^ were addressed in the present study, including temporality and biological gradient. An especially important contribution is the evidence that the effects of high PWL are reversible following reduction or removal of exposure.

From a prevention perspective, reducing cumulative exposure to physically demanding tasks across working life is likely to be beneficial. While the present study does not address specific interventions, such reductions may be achieved through ergonomic improvements, reskilling and other organisational measures limiting prolonged PWL.

## Conclusions

In both men and women, changing from higher to lower exposure to PWL was associated with lower risk of MSD work disability, and changing from lower to higher exposure was associated with higher risks. Reducing life-course exposure to heavy PWL could improve people’s labour-market prospects associated with musculoskeletal health and function. These results underscore the importance of preventive strategies aimed at reducing time spent on physically demanding work tasks, although future research is needed to identify the most effective approaches.

## Supplementary material

10.1136/oemed-2026-111034 online supplemental table 1

## Data Availability

Data may be obtained from a third party and are not publicly available.
