# Supplementary material for: Change in physical workload and the risk of work disability due to musculoskeletal disorders: a register-based study of 1 703 935 workers in Sweden
Source: Occup Environ Med. 2026 Jul 28;83(5):e111034. doi: 10.1136/oemed-2026-111034 (PMC13539868; doi:10.1136/oemed-2026-111034)
Supplement: Supplementary file 1 [file oemed-83-5-s001.docx]

**Supplementary Tables**

**Table S1. Characterization of the cohort according to all covariates in relation to occupational physical workload (PWL) measured in 1990 and in 2005.**

| **PWL 1990** | **Low** | | | | **Mid** | | | | **High** | | | |
| --- | --- | --- | --- | --- | --- | --- | --- | --- | --- | --- | --- | --- |
| **PWL 2005** | **Low** | **Med** | **High** | **Missing** | **Low** | **Med** | **High** | **Missing** | **Low** | **Med** | **High** | **Missing** |
| **Women N (%)** | 222,531  (77.0%) | 36,254  (13.0%) | 15,304  (5.3%) | 14,904  (5.2%) | 93,610  (35%) | 125,590  (46%) | 36,293  (13%) | 15,109  (5.6%) | 24,806  (10%) | 35,249  (15%) | 161,302  (66%) | 21,549  (8.9%) |
| **Characteristics %** |  |  |  |  |  |  |  |  |  |  |  |  |
| Age |  |  |  |  |  |  |  |  |  |  |  |  |
| 44-49 | 23.7 | 25.8 | 27.9 | 21.4 | 31.6 | 31.9 | 33.7 | 24.5 | 46.6 | 38.4 | 30.2 | 28.1 |
| 50-59 | 54.9 | 54.8 | 53.9 | 46.5 | 52.2 | 52.0 | 50.0 | 44.8 | 43.6 | 46.8 | 50.8 | 47.2 |
| 60-64 | 21.4 | 19.4 | 18.2 | 32.1 | 16.2 | 16.1 | 16.3 | 30.7 | 9.8 | 14.8 | 19.0 | 24.7 |
| Education |  |  |  |  |  |  |  |  |  |  |  |  |
| ≤9 | 8.4 | 10.4 | 18.5 | 14.4 | 8.9 | 8.1 | 21.6 | 22.4 | 12.3 | 25.4 | 29.4 | 34.7 |
| 10-11 | 24.8 | 29.5 | 49.6 | 31.3 | 24.7 | 34.0 | 55.0 | 36.4 | 34.9 | 42.0 | 56.4 | 36.7 |
| 12 | 11.4 | 10.2 | 15.9 | 16.0 | 13.6 | 6.5 | 12.5 | 13.8 | 14.6 | 10.6 | 8.9 | 18.3 |
| 13-14 | 14.4 | 30.8 | 10.0 | 16.8 | 22.7 | 35.7 | 8.1 | 15.6 | 15.2 | 11.8 | 3.3 | 5.5 |
| ≥15 | 41.0 | 19.1 | 6.0 | 21.5 | 30.1 | 15.7 | 2.8 | 11.8 | 23.0 | 10.2 | 2.0 | 4.8 |
| Marital status |  |  |  |  |  |  |  |  |  |  |  |  |
| Married | 62.6 | 61.7 | 54.3 | 59.5 | 63.1 | 63.5 | 56.4 | 60.2 | 58.9 | 59.5 | 57.7 | 61.2 |
| Unmarried | 16.1 | 16.7 | 19.0 | 16.6 | 16.1 | 17.0 | 18.7 | 16.8 | 18.2 | 18.0 | 19.3 | 16.8 |
| Divorced | 18.5 | 18.7 | 23.0 | 19.9 | 18.3 | 16.9 | 21.5 | 19.0 | 20.7 | 19.5 | 19.1 | 17.9 |
| Widowed | 2.8 | 2.9 | 3.7 | 4.0 | 2.6 | 2.6 | 3.4 | 4.0 | 2.2 | 3.0 | 3.9 | 4.1 |
| Foreign born | 6.9 | 7.4 | 10.2 | 11.1 | 6.5 | 7.0 | 11.1 | 11.0 | 9.9 | 9.8 | 11.8 | 13.8 |
| Parents’ occupation |  |  |  |  |  |  |  |  |  |  |  |  |
| Unskilled manual | 23.2 | 25.6 | 31.7 | 22.3 | 24.8 | 27.5 | 32.4 | 25.4 | 28.6 | 31.5 | 34.8 | 29.2 |
| Skilled manual | 19.3 | 20.9 | 23.3 | 18.6 | 20.7 | 22.2 | 23.7 | 19.0 | 21.9 | 23.9 | 23.3 | 21.5 |
| Assistant non-manual | 12.4 | 11.6 | 8.8 | 12.5 | 12.3 | 10.9 | 7.7 | 11.4 | 10.0 | 9.1 | 6.0 | 7.9 |
| Intermediate non-manual | 20.4 | 18.1 | 12.4 | 18.9 | 19.1 | 16.1 | 10.6 | 16.0 | 15.3 | 11.7 | 7.8 | 9.7 |
| Higher non-manual | 8.1 | 6.0 | 3.3 | 8.3 | 6.2 | 4.9 | 2.3 | 6.6 | 4.4 | 2.7 | 1.6 | 3.1 |
| Farmer | 7.1 | 7.8 | 7.7 | 5.4 | 7.8 | 8.5 | 9.0 | 6.9 | 7.4 | 8.6 | 10.8 | 11.5 |
| No information | 9.5 | 10.0 | 12.8 | 14.1 | 9.1 | 9.9 | 14.3 | 14.7 | 12.4 | 12.5 | 15.7 | 17.1 |
| Previous DP due to other causes | 4.0 | 4.2 | 4.0 | 4.7 | 3.3 | 4.3 | 4.2 | 4.8 | 4.0 | 3.8 | 4.2 | 5.6 |
| Unemployment days between 1996 and 2005 |  |  |  |  |  |  |  |  |  |  |  |  |
| 0 | 83.4 | 73.3 | 46.5 | 54.2 | 79.1 | 83.6 | 57.5 | 57.6 | 60.0 | 63.5 | 74.7 | 58.8 |
| 1-365 | 10.4 | 15.6 | 28.3 | 16.0 | 13.6 | 11.4 | 26.1 | 16.5 | 24.1 | 22.7 | 15.6 | 15.3 |
| 366-730 | 3.8 | 6.3 | 13.7 | 12.1 | 4.6 | 3.2 | 9.8 | 11.2 | 9.4 | 8.3 | 5.6 | 10.7 |
| >730 | 2.4 | 4.8 | 11.5 | 17.7 | 2.7 | 1.8 | 6.6 | 14.7 | 6.5 | 5.5 | 4.1 | 15.2 |
| Sick leave days between 1996 and 2005 |  |  |  |  |  |  |  |  |  |  |  |  |
| 0 | 49.6 | 42.9 | 34.1 | 48.8 | 47.8 | 40.4 | 32.4 | 45.5 | 40.9 | 37.7 | 32.9 | 43.1 |
| 1-365 | 39.5 | 45.1 | 52.5 | 38.1 | 41.3 | 46.4 | 53.3 | 40.9 | 45.7 | 49.2 | 54.0 | 43.6 |
| 366-730 | 5.2 | 5.9 | 7.1 | 5.6 | 5.5 | 6.3 | 7.7 | 6.0 | 6.7 | 6.9 | 7.3 | 6.1 |
| >730 | 5.7 | 6.1 | 6.3 | 7.5 | 5.4 | 6.9 | 6.5 | 7.6 | 6.7 | 6.2 | 5.8 | 7.2 |
| **Men (N)** | 233,647  (75%) | 38,687  (12%) | 16,781  (5.4%) | 23,287  (7.3%) | 84,333  (29%) | 113,163  (39%) | 59,739  (20%) | 35,308  (12%) | 22,837  (7.7%) | 48,559  (16%) | 178,560  (60%) | 46,533  (16%) |
| **Characteristics %** |  |  |  |  |  |  |  |  |  |  |  |  |
| Age |  |  |  |  |  |  |  |  |  |  |  |  |
| 44-49 | 25.0 | 24.8 | 24.1 | 17.7 | 30.9 | 28.9 | 33.1 | 26.0 | 42.7 | 36.5 | 32.5 | 29.9 |
| 50-59 | 52.9 | 54.0 | 53.5 | 49.2 | 51.6 | 52.4 | 50.6 | 48.8 | 45.6 | 49.0 | 50.8 | 49.7 |
| 60-64 | 22.1 | 21.2 | 22.4 | 33.1 | 17.5 | 18.7 | 16.3 | 25.2 | 11.7 | 14.5 | 16.7 | 20.4 |
| Education |  |  |  |  |  |  |  |  |  |  |  |  |
| ≤9 | 4.8 | 12.8 | 25.6 | 11.3 | 14.3 | 26.0 | 35.6 | 29.8 | 23.5 | 35.3 | 42.8 | 41.8 |
| 10-11 | 12.5 | 29.7 | 36.3 | 19.1 | 26.5 | 34.0 | 45.2 | 33.9 | 38.7 | 44.7 | 44.5 | 40.2 |
| 12 | 17.9 | 21.2 | 19.8 | 22.7 | 19.4 | 15.4 | 13.3 | 17.7 | 15.2 | 11.6 | 8.8 | 10.6 |
| 13-14 | 20.5 | 23.5 | 12.9 | 19.0 | 19.0 | 16.9 | 4.5 | 10.9 | 11.6 | 5.9 | 2.4 | 3.7 |
| ≥15 | 44.3 | 12.8 | 5.2 | 27.9 | 20.8 | 7.7 | 1.4 | 7.7 | 11.0 | 2.5 | 1.5 | 3.7 |
| Marital status |  |  |  |  |  |  |  |  |  |  |  |  |
| Married | 70.6 | 61.2 | 61.3 | 61.5 | 66.8 | 58.9 | 52.8 | 53.9 | 62.7 | 54.4 | 53.6 | 51.1 |
| Unmarried | 15.0 | 20.3 | 20.0 | 18.6 | 17.0 | 23.4 | 29.0 | 25.0 | 20.5 | 26.8 | 29.4 | 29.3 |
| Divorced | 13.3 | 17.3 | 17.5 | 18.4 | 15.2 | 16.6 | 17.2 | 19.8 | 15.9 | 17.8 | 15.9 | 18.5 |
| Widowed | 1.1 | 1.2 | 1.2 | 1.5 | 1.0 | 1.1 | 1.0 | 1.3 | 0.9 | 1.0 | 1.1 | 1.1 |
| Foreign born | 5.5 | 7.3 | 8.4 | 8.9 | 5.4 | 7.7 | 11.2 | 12.4 | 9.0 | 11.3 | 9.6 | 13.9 |
| Parents’ occupation |  |  |  |  |  |  |  |  |  |  |  |  |
| Unskilled manual | 20.8 | 27.3 | 30.6 | 21.0 | 25.8 | 31.4 | 34.2 | 26.7 | 29.1 | 34.7 | 34.2 | 28.5 |
| Skilled manual | 18.2 | 21.6 | 24.1 | 18.1 | 20.8 | 22.6 | 24.2 | 19.9 | 23.7 | 23.5 | 23.9 | 21.1 |
| Assistant non-manual | 13.3 | 11.7 | 8.5 | 12.7 | 14.0 | 10.8 | 7.5 | 12.1 | 9.3 | 6.7 | 5.5 | 5.8 |
| Intermediate non-manual | 24.2 | 18.1 | 13.7 | 21.1 | 19.2 | 13.3 | 9.8 | 13.2 | 14.1 | 9.4 | 7.7 | 7.9 |
| Higher non-manual | 9.8 | 5.3 | 3.2 | 9.2 | 6.3 | 3.8 | 1.8 | 4.8 | 4.0 | 2.1 | 1.4 | 2.2 |
| Farmer | 5.6 | 5.6 | 8.3 | 6.0 | 5.7 | 7.0 | 7.8 | 7.4 | 8.1 | 9.1 | 14.1 | 17.1 |
| No information | 8.1 | 10.4 | 11.6 | 11.9 | 8.2 | 11.1 | 14.7 | 15.9 | 11.7 | 14.5 | 13.2 | 17.4 |
| Previous DP due to other causes | 1.2 | 2.4 | 1.9 | 2.8 | 1.4 | 2.2 | 2.3 | 3.2 | 1.5 | 2.7 | 2.3 | 3.4 |
| Unemployment days between 1996 and 2005 |  |  |  |  |  |  |  |  |  |  |  |  |
| 0 | 87.6 | 75.6 | 63.7 | 63.8 | 81.6 | 80.4 | 69.0 | 57.6 | 69.7 | 66.5 | 67.6 | 59.1 |
| 1-365 | 7.7 | 13.2 | 18.4 | 14.1 | 11.2 | 11.2 | 16.0 | 16.4 | 17.6 | 16.9 | 16.1 | 15.4 |
| 366-730 | 2.9 | 5.8 | 9.1 | 9.0 | 4.3 | 4.6 | 7.4 | 10.7 | 6.9 | 8.2 | 7.7 | 9.5 |
| >730 | 1.8 | 5.4 | 8.8 | 13.1 | 2.9 | 3.8 | 7.6 | 15.3 | 5.8 | 8.4 | 8.6 | 16.0 |
| Sick leave days between 1996 and 2005 |  |  |  |  |  |  |  |  |  |  |  |  |
| 0 | 72.6 | 58.8 | 51.2 | 60.8 | 67.1 | 54.7 | 47.5 | 52.0 | 58.5 | 47.3 | 45.2 | 44.5 |
| 1-365 | 23.3 | 34.0 | 41.0 | 30.8 | 27.9 | 38.3 | 44.7 | 37.2 | 34.2 | 43.9 | 47.0 | 44.1 |
| 366-730 | 2.2 | 3.9 | 4.6 | 3.8 | 2.8 | 3.9 | 4.6 | 5.1 | 4.1 | 5.1 | 4.7 | 5.9 |
| >730 | 1.9 | 3.3 | 3.2 | 4.6 | 2.2 | 3.1 | 3.2 | 5.7 | 3.2 | 3.7 | 3.1 | 5.5 |

**Table S2. Association between levels of physical workload (PWL) in 1990 and 2005 and risk of work disability due to a musculoskeletal disorder (MSD) among women and men, stratified by age and PWL in 1990. Those stable in low, mid, or high exposure between 1990 and 2005 are used as the reference category. Hazard ratios (HR) and 95% confidence intervals (CI).**

|  |  | **Age in 1990** | | | | | |
| --- | --- | --- | --- | --- | --- | --- | --- |
|  |  | **29-34 years** | | **35-44 years** | | **45-49 years** | |
|  |  | **Adjusted** | | **Adjusted** | | **Adjusted** | |
| **PWL**  **1990** | **PWL**  **2005** | **MSD %** | **95% CI** | **MSD %** | **95% CI** | **MSD %** | **95% CI** |
| **Women** |  |  |  |  |  |  |  |
| Low | = | 1.1 | 1 | 1.3 | 1 | 0.8 | 1 |
|  | +1 | 1.8 | 1.28 (1.08, 1.53) | 2.2 | 1.36 (1.22, 1.52) | 1.5 | 1.53 (1.22, 1.90) |
|  | +2 | 3.1 | 1.41 (1.15, 1.72) | 3.9 | 1.70 (1.49, 1.93) | 3.3 | 2.65 (2.07, 3.38) |
|  | Missing | 2.4 | 1.22 (0.94, 1.57) | 2.7 | 1.41 (1.20, 1.65) | 1.0 | 1.19 (0.87, 1.63) |
| Med | = | 2.1 | 1 | 2.7 | 1 | 2.3 | 1 |
|  | -1 | 1.4 | 0.81 (0.72, 0.91) | 1.6 | 0.72 (0.66, 0.79) | 1.0 | 0.49 (0.41, 0.59) |
|  | +1 | 3.8 | 1.21 (1.08, 1.37) | 4.4 | 1.18 (1.08, 1.29) | 4.0 | 1.52 (1.28, 1.80) |
|  | Missing | 3.7 | 1.23 (1.02, 1.49) | 3.7 | 1.19 (1.04, 1.37) | 1.3 | 0.73 (0.56, 0.97) |
| High | = | 4.1 | 1 | 4.9 | 1 | 4.2 | 1 |
|  | -1 | 3.1 | 0.86 (0.77, 0.95) | 3.6 | 0.76 (0.70, 0.83) | 3.0 | 0.75 (0.64, 0.89) |
|  | -2 | 2.2 | 0.69 (0.60, 0.79) | 2.5 | 0.58 (0.51, 0.66) | 1.4 | 0.37 (0.26, 0.52) |
|  | Missing | 4.6 | 0.97 (0.85, 1.10) | 4.1 | 0.88 (0.79, 0.97) | 2.0 | 0.63 (0.52, 0.77) |
| **Men** |  |  |  |  |  |  |  |
| Low | = | 0.3 | 1 | 0.4 | 1 | 0.3 | 1 |
|  | +1 | 1.1 | 1.82 (1.40, 2.38) | 1.2 | 1.62 (1.38, 1.91) | 0.8 | 1.75 (1.28, 2.39) |
|  | +2 | 1.7 | 2.14 (1.57, 2.92) | 1.9 | 2.02 (1.67, 2.44) | 1.9 | 3.31 (2.42, 4.53) |
|  | Missing | 1.1 | 1.78 (1.25, 2.53) | 1.2 | 1.60 (1.31, 1.96) | 0.6 | 1.46 (1.02, 2.09) |
| Med | = | 1.2 | 1 | 1.5 | 1 | 1.4 | 1 |
|  | -1 | 0.6 | 0.76 (0.63, 0.92) | 0.7 | 0.70 (0.61, 0.79) | 0.5 | 0.53 (0.41, 0.68) |
|  | +1 | 1.7 | 1.10 (0.95, 1.28) | 2.4 | 1.24 (1.12, 1.37) | 2.2 | 1.31 (1.09, 1.56) |
|  | Missing | 2.2 | 1.20 (0.99, 1.43) | 2.3 | 1.16 (1.02, 1.31) | 1.5 | 1.06 (0.86, 1.32) |
| High | = | 1.9 | 1 | 2.9 | 1 | 3.0 | 1 |
|  | -1 | 1.8 | 0.92 (0.81, 1.04) | 2.2 | 0.75 (0.68, 0.82) | 2.1 | 0.70 (0.59, 0.84) |
|  | -2 | 1.1 | 0.74 (0.60, 0.91) | 1.5 | 0.55 (0.47, 0.65) | 1.7 | 0.70 (0.52, 0.95) |
|  | Missing | 2.6 | 1.10 (0.98, 1.24) | 3.2 | 0.96 (0.88, 1.04) | 2.2 | 0.76 (0.65, 0.88) |

= unchanged; -1 decreased by 1 level; -2 decreased by 2 levels; +1 increased by 1 level; +2 increased by 2 levels

Adjusted for age, education, marital status, country of birth, early life SEP, previous DP due to other causes, unemployment days 1996-2005, and sickness absence days 1996-2005
